# FGF1 induces resistance to chemotherapy in ovarian granulosa tumor cells through regulation of p53 mitochondrial localization

**DOI:** 10.1038/s41389-018-0033-y

**Published:** 2018-02-21

**Authors:** Sevasti Manousakidi, Arnaud Guillaume, Caroline Pirou, Sylvina Bouleau, Bernard Mignotte, Flore Renaud, Nathalie Le Floch

**Affiliations:** 1 Laboratoire de Génétique et Biologie Cellulaire, EA4589, Université de Versailles Saint-Quentin-en-Yvelines / Université Paris-Saclay, Montigny-Le-Bretonneux, France; 2 LGBC, Ecole Pratique des Hautes Etudes EPHE, PSL Research University, Montigny-Le-Bretonneux, France; 3 IUT de Vélizy/Rambouillet, Université de Versailles Saint-Quentin-en-Yvelines / Université Paris-Saclay, 19 allée des Vignes, Rambouillet, France

## Abstract

Ovarian cancer remains associated with a high mortality rate and relapse is too frequently seen after chemotherapeutic treatment of granulosa cell tumors (GCTs) or epithelial ovarian cancers (EOCs). It is thus of major importance to progress in the knowledge of the molecular mechanisms underlying chemoresistance of ovarian tumors. Overexpression of Fibroblast Growth Factor 1 (FGF1) is observed in various cancers, correlates with poor survival and could be responsible for resistance to platinum-based chemotherapy of serous ovarian cancers. How FGF1 promotes escape to chemotherapy remains unknown. In previous studies, we showed that FGF1 inhibits p53 transcriptional activities, leading to increased cell survival of neuronal or fibroblast cell lines. In this study, we show that FGF1 favors survival of COV434 cells upon treatment with etoposide and cisplatin, two common chemotherapeutic molecules used for ovarian cancer. Etoposide and cisplatin induced mitochondrial depolarization, cytochrome c release and caspase activation in COV434 cells. Overexpression of FGF1 counteracts these events and thus allows increased survival of ovarian cells. In this study, FGF1 had little effect on p53 stability and transcriptional activities. Etoposide induced p21 expression as expected, but p21 protein levels were even increased in the presence of FGF1. Using RNA interference, we showed that p21 exerts an anti-apoptotic activity in COV434 cells. However abrogating this activity was not sufficient to restore cell death of FGF1-overexpressing cells. We also show for the first time that p53 mitochondrial pathway is involved in the cell death of COV434 cells. Indeed, p53 accumulates at mitochondria upon etoposide treatment and inhibition of p53 mitochondrial localization using pifithrin-µ inhibits apoptosis of COV434 cells. FGF1 also decreases mitochondrial accumulation of p53 induced by etoposide. This constitutes a novel mechanism of action for FGF1 to promote cell survival in response to chemotherapy.

## Introduction

There were an estimated 65,500 new cases of ovarian cancer in 2012 in Europe with 42,700 deaths^1^. It ranks fifth as the cause of cancer death in women and is the most deadly gynecological cancer due to late stage diagnosis^2^. Ovarian cancer is not a single disease, but a group of tumors classified depending on the cells it involves. Thereby, there are ovarian epithelial tumors, sex cord-stromal tumors (implicating granulosa and theca cells) and germ cell tumors^3^.

Advanced ovarian epithelial cancer patients undergo surgery, in order to reduce all macroscopic visible disease. Early and advanced stage epithelial cancers are treated with a combination therapy of platinum and taxane. Unfortunately, approximately 70% of the patients present a relapse during the first 3 years^4^. The most common therapy regimen for sex cord- stromal ovarian tumors is the combination of bleomycin, etoposide and cisplatin (BEP)^5^. Even though these tumors show a good response rate after BEP treatment, a high relapse rate is observed several months after the completion of the treatment^6^.

Overexpression of Fibroblast Growth Factor 1 (FGF1) has been linked to high grade serous ovarian tumors and poor survival^7,8^. Furthermore, FGF1 has been associated with tumor growth in nude mice injected with ovarian cells overexpressing FGF1^9^. In ovarian epithelial cisplatin-resistant cell lines overexpressing FGF1, its knock-down by shRNA, restores sensitivity to cisplatin^8^.

FGF1 belongs to the FGF family that counts 22 members^10,11^. FGF1 regulates cell proliferation, differentiation and survival^12–19^. FGF1 acts through FGFR–dependent or FGFR–independent pathways^16,19–21^. Indeed, FGF1 is mainly intracellular under physiological conditions and secreted only under specific stress conditions^22–24^. Whereas FGF1 has been shown to interact with intracellular proteins such as CK2, FIBP, p34, nucleolin, and p53^18,21,25–28^, its intracellular activities are not fully understood. Nevertheless, FGF1 intracellular activities are crucial for cell survival since FGF1 represses the pro-apoptotic activity of p53. We previously showed in rat embryonic fibroblasts and pheochromocytoma PC12 cell line that FGF1 promotes p53 degradation and inhibits both p53 phosphorylation on serine 15 and p53 transcriptional activities^16,17^. We also showed that FGF1 interacts with p53 in PC12 cells^18^.

p53 is a key regulator of apoptosis^29^. Its ability to induce apoptosis is mediated by the transactivation of pro-apoptotic genes such as *Bax*^30^, *NOXA* and *PUMA*^31^. p53 also triggers apoptosis by relocating at the mitochondrion. Indeed, p53 mitochondrial translocation allows its interaction with both the anti-apoptotic proteins BCL-X_L_ and Bcl-2, leading to their inhibition^32^, and the pro-apoptotic proteins Bax and BAK, provoking their activation^33,34^.

As FGF1 can inhibit p53-dependent apoptosis, we hypothesized that FGF1 could affect the apoptotic response to etoposide (an activator of p53-dependent apoptosis) in ovarian tumor cells. We tested our hypothesis in the ovarian granulosa cell line COV434 that expresses wild-type p53 protein^35^. In the present study, we showed that FGF1 is able to attenuate etoposide and cisplatin-induced apoptosis in COV434 cells. Under etoposide treatment, FGF1 only shows a moderate impact on p53 stability or activation and p53 transcriptional activities do not appear to be involved in COV434 cells apoptosis. However, p53 mitochondrial activities are important for COV434 cells apoptosis and FGF1 regulates p53 mitochondrial translocation.

## Results

### FGF1 overexpression protects COV434 ovarian granulosa cells from etoposide-induced apoptosis

We first determined whether FGF1 overexpression could be sufficient to induce resistance to chemotherapeutic agents such as cisplatin or etoposide in ovarian cells. We thus established stable cell lines constitutively overexpressing FGF1 (COV434-FGF1). To induce p53-dependent cell death, we used the well-known genotoxic stress inducer etoposide. Among the hallmarks of apoptosis, we monitored (i) the decrease of the inner mitochondrial membrane potential (ΔΨm) that reflects mitochondrial depolarization during apoptosis, (ii) cytochrome c release arising from mitochondrial outer membrane permeabilization (MOMP) and (iii) caspases activation.

**Fig. 1:**
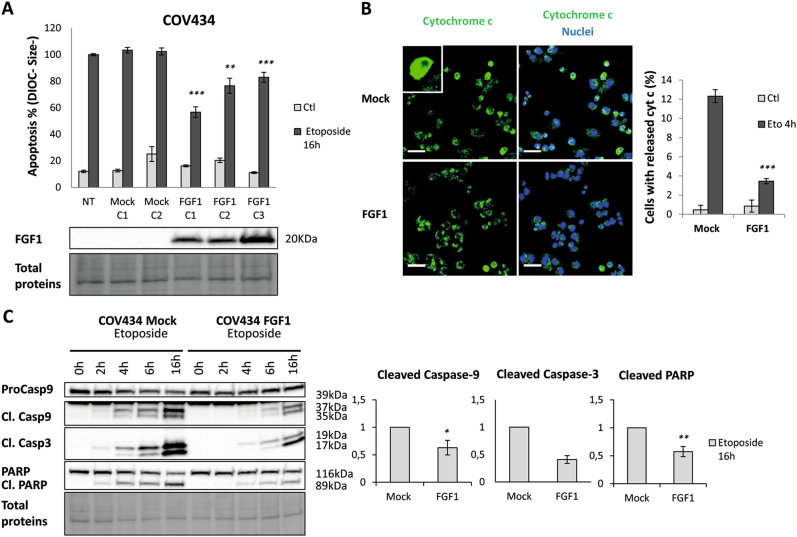
FGF1 overexpression protects COV434 cells from etoposide-induced apoptosis. **a** Upper panel: Average flow cytometry quantification of apoptotic cells characterized by their low DIOC staining and cell condensation (DIOC^-^, Size^-^) ± SEM for 3 experiments done in triplicate. Non-transfected COV434 (NT), two COV434-Mock clonal cell lines and three COV434-FGF1 clonal cell lines were treated with etoposide (25 µg/mL) for 16 h, or not treated (Ctl), The t-tests compare to NT Eto. Lower panel: FGF1 levels in non-transfected, mock and FGF1 overexpressing clones using western blot analysis. Total proteins are visualized with the Biorad stain free system. **b** Immunofluorescence study for cytochrome c release. COV434-Mock C1 and -FGF1 C1 cells were treated with 25 µg/mL etoposide for 4 h. Cells were stained with an anti-cytochrome c antibody (green) and TO-PRO-3 (blue) to visualize nuclei (left panels). Scale bar represents 40 µm. The histogram presents the average percentages ± SEM for 3 independent experiments of cells exhibiting cytochrome c release (right panel). The t-test compares to Mock cells similarly treated. **c** Upper panel: Western blot analysis of total proteins for procaspase-9, cleaved caspase-9 and −3 and PARP levels. COV434-Mock and -FGF1 cells were treated or not (0 h) with 25 µg/mL etoposide for 2, 4, 6, or 16 h. Lower panel: histograms present the average fold-change decrease of cleaved caspase-9, cleaved caspase-3 and cleaved PARP in COV434-FGF1 cells ± SEM from pooled results of three FGF1 overexpressing clones (*n* = 8). Two-tailed unpaired t-tests results are shown as * for *P* ≤ 0.05, ** for *P* ≤ 0.01 and *** for *P* ≤ 0.001

Cell condensation and loss of ΔΨm reflected by low DiOC_6_(3) staining were first examined by flow cytometry analysis. Following a 16 h-long etoposide treatment, the percentage of COV434-FGF1 cells with small size and low ΔΨm (apoptotic cells) was significantly lower than for parental or mock-transfected COV434 cells (Fig. 1a). Similar results were obtained using cisplatin instead of etoposide (Supplementary Fig. S1A). Therefore, FGF1 overexpression partially inhibits etoposide- and cisplatin-induced apoptosis in COV434 cells.

Release of cytochrome c from intermembrane mitochondrial space was then studied using immunofluorescence. Following 4 h of etoposide treatment, FGF1 significantly decreased the release of cytochrome c (Fig. 1b). This suggests that FGF1 inhibits MOMP during etoposide-induced cell death.

We further examined the effect of FGF1 overexpression on the cleavage of procaspase-9 and procaspase−3 and the caspase target PARP following an etoposide treatment. In COV434-FGF1 cells, the cleavage of these apoptotic markers is delayed and less pronounced than in COV434-Mock cells (Fig. 1c). Similar results were obtained with cisplatin instead of etoposide (Supplementary Figs. S1B, S1C).

Therefore, FGF1 overexpression in COV434 cells is able to prevent a decrease in ΔΨm, cytochrome c release and the subsequent activation of caspases arising during etoposide and cisplatin-induced apoptosis. These results suggest that FGF1 acts either upstream or at the mitochondrial level to promote survival.

### FGF1 overexpression attenuates the etoposide-induced G2/M cell cycle arrest

As etoposide provokes an S phase delay and a G2 phase accumulation of treated cells^36^, we explored the effect of FGF1 overexpression on cell cycle. This was done by cytometry analysis after Hoechst 33342 staining.

**Fig. 2:**
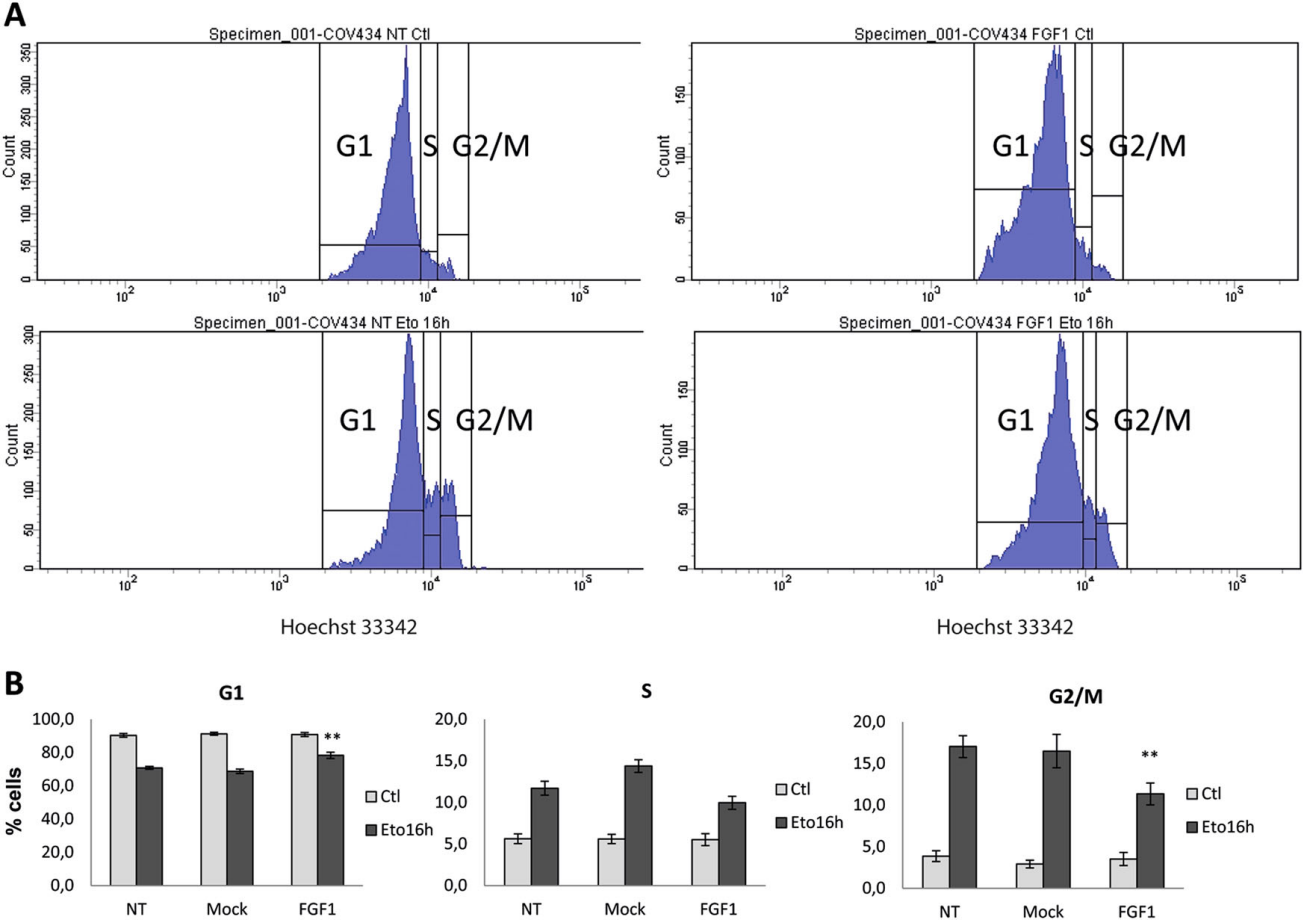
FGF1 overexpression attenuates the etoposide-induced G2/M cell cycle arrest. Non-transfected (NT), mock and FGF1-overexpressing COV434 cells were treated with etoposide for 16 h, or untreated (Ctl). DNA was stained with Hoechst 33342 and cellular content was analyzed by flow cytometry. **a** Cytograms showing cell cycle phase distribution (G1, S, G2/M). **b** The histograms show the average percentages of cells in each cell cycle phase ± SEM. Results for 4 independent experiments in replicate (*n* = 10). T-tests compare to NT Eto. *P ≤ 0.05, **P ≤ 0.01, ***P ≤ 0.001

Distribution of cells in the different cell cycle phases was similar for COV434 (COV434-NT), COV434-Mock and COV434-FGF1 cells in the absence of etoposide (Fig. 2a, b). After 16 h of etoposide treatment, we observed a decrease of the percentage of cells in G1 phase and an increase of the percentage of cells in S and G2/M phases in all cell populations as expected. Interestingly, a significantly lower proportion of COV434-FGF1 cells accumulated in G2/M phases compared to COV434-NT and -Mock cells. In conclusion, FGF1 partly inhibits the etoposide-induced cell cycle arrest at G2/M phases in COV434 cells.

### The FGF1 anti-apoptotic activity involves mainly a FGFR-independent pathway

**Fig. 3:**
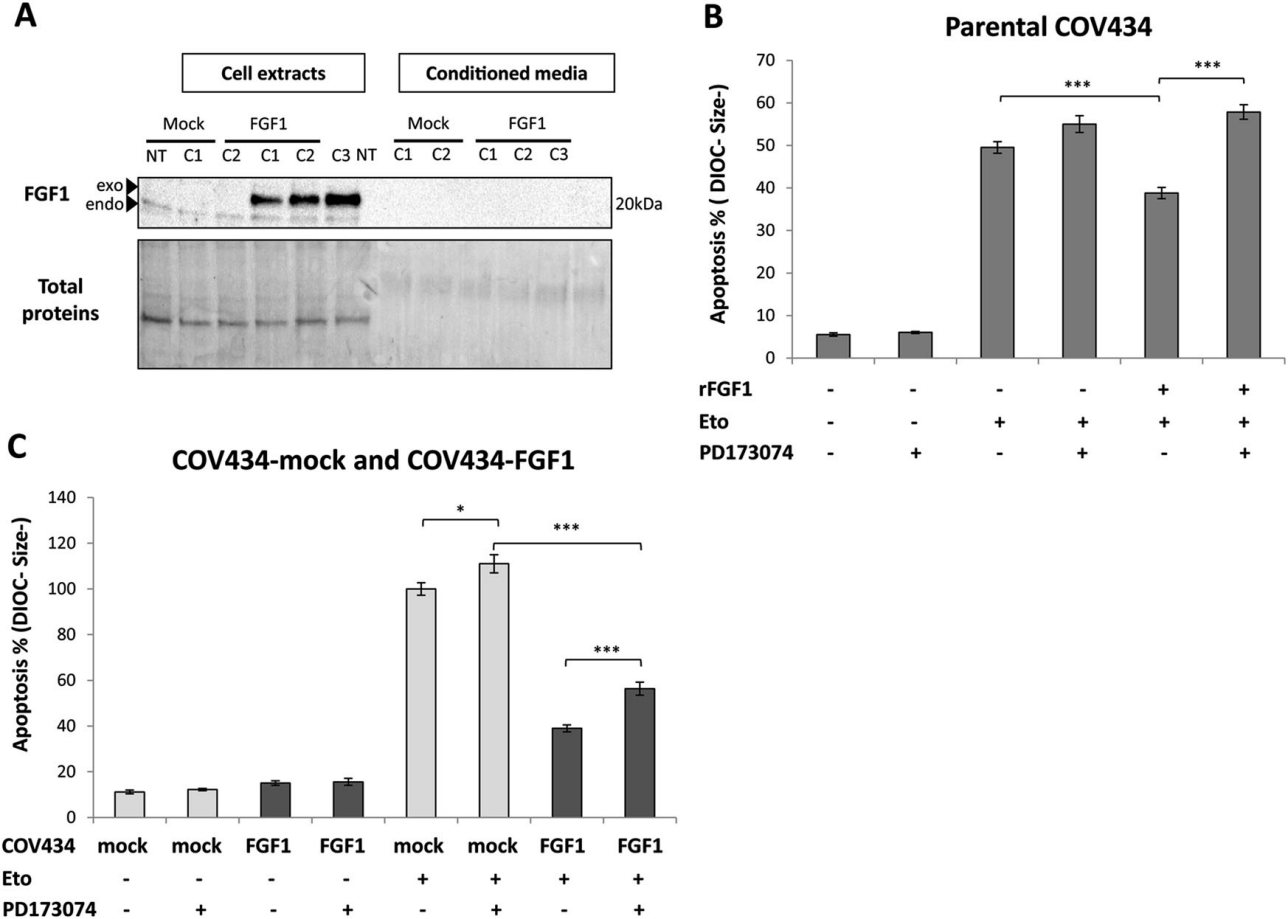
Both FGFR-dependent and FGFR-independent pathways are involved in FGF1 anti-apoptotic activity. **a** Western blot analysis for FGF1 levels in total extracts and conditioned media of non-transfected COV434 (NT), COV434-Mock and COV434-FGF1 cells. Endogenous FGF1 is detected in all total extracts whereas exogenous FGF1-V5-His is seen only in FGF1-overexpressing cells as expected. **b** Average apoptosis rates ± SEM for 3 experiments done in triplicate measured by flow cytometry of COV434-Mock cells. Cells were pretreated or not with the FGFR1/3 inhibitor PD173074 (25 nM for one hour), followed or not by a treatment with 50 ng/mL of recombinant FGF1 (rFGF1) supplemented with 10 µg/mL heparin for 24 h. On the next day, these treatments were renewed adding or not etoposide for 16 h. **c** Average apoptosis rates ± SEM for 3 experiments done in triplicate measured by flow cytometry of COV434-Mock and COV434-FGF1 cells pretreated or not with 25 nM PD173074 for 24 h, and treated or not with etoposide (25 µg/mL for 16 h). Two-tailed *t*-tests are indicated by * for *P* ≤ 0.05, ** for *P* ≤ 0.01, and *** for *P* ≤ 0.001

FGF1 lacks a secretion signal peptide and is not secreted under basal conditions. As expected, affinity chromatography of conditioned media using heparin sepharose showed that FGF1 is not detected in the culture medium of COV434-FGF1 cells in the absence of etoposide (Fig. 3a). Under stress conditions such as hypoxia or heat shock, FGF1 can be secreted through non-canonical pathways and activate FGFRs on neighboring cells^37^. Therefore, we wanted to evaluate the contribution of the FGFR-dependent and FGFR-independent pathways in the anti-apoptotic activity of FGF1. For this purpose, the FGFR-dependent pathway was inhibited using the FGFR1/3 inhibitor PD173074. The efficiency of this inhibitor towards rFGF1 activity was confirmed. Indeed, recombinant FGF1 (rFGF1) added in the culture medium partly protected COV434 cells from etoposide-induced cell death and the addition of PD173074 abrogated this anti-apoptotic activity (Fig. 3b). Interestingly, PD173074 only partially reversed FGF1-induced resistance to etoposide-induced apoptosis in COV434-FGF1 cells (Fig. 3c). These data suggest that overexpressed FGF1 acts mainly through FGFR-independent pathways to exert its anti-apoptotic activity in COV434 cells.

### FGF1 does not affect p53 transcriptional activities in the COV434 ovarian cell line

Etoposide is known to activate the pro-apoptotic p53 protein. In our previous studies, we have shown that FGF1 is able to regulate p53-dependent apoptosis by decreasing p53 stability, phosphorylation and transcriptional activities in fibroblast and neuronal cells^16–19^. We thus wondered whether FGF1 could exert its anti-apoptotic activities in a similar way in COV434 cells. First, we examined p53 protein levels and phosphorylation at serine 15, a post-translational modification crucial for its transcriptional activity^38^.

**Fig. 4:**
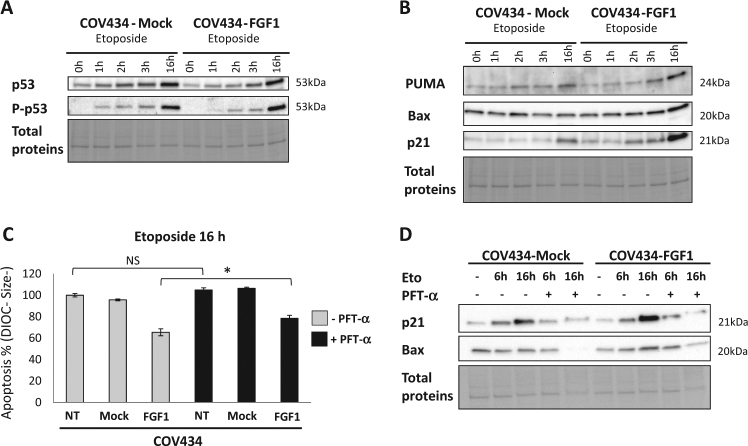
p53 transcriptional-dependent activities are dispensable for the apoptosis of the COV434 ovarian cell line. **a** COV434-Mock and COV434-FGF1 cells were treated or not with 25 µg/mL etoposide for 1, 2, 3 or 16 h. Total protein extracts were analyzed for p53 and Ser15-phosphorylated p53 by western blotting. **b** COV434 Mock and COV434-FGF1 cells were treated or not with etoposide for 1, 2, 3 or 16 h. Total proteins were analyzed for PUMA, Bax and p21 protein levels by western blotting. **c** Average apoptosis rates ± SEM for 2 experiments done in triplicate measured by flow cytometry of non-transfected COV434 (NT), Mock and FGF1 cells treated with etoposide for 16 h. Cells were pretreated or not for 90 min with the p53 transcriptional inhibitor pifithrin-alpha (PFT-α, 30 µM). **d** Western blot analysis of p21 and Bax protein levels in COV434 Mock and FGF1 cells pretreated with PFT-α (30 µM for 90 min), followed by an etoposide (25 µg/mL) treatment for 6 or 16 h. Two-tailed *t*-tests results are shown by * for *P* ≤ 0.05, ** for *P* ≤ 0.01, and *** for *P* ≤ 0.001

Without etoposide, there is no significant difference in p53 protein levels between COV434-Mock and -FGF1 cells. After 2 or 3 h of etoposide treatment, we observed significantly lower p53 levels in COV434-FGF1 compared to Mock cells but no difference after 16 h of treatment (Fig. 4a and Supplementary Fig. S2A). Even though p53 levels are lower in COV434-FGF1 at 2 and 3 h, levels of p53 phosphorylated at serine 15 are not lower and even significantly higher after 16 h of etoposide treatment (Fig. 4a and Supplementary Fig. S2B).

We next examined the protein levels of p53 transcriptional targets such as *PUMA*, *Bax*, *p21,* and *TIGAR*. We have previously shown that *Bax* and *PUMA* transactivation by p53 is attenuated in the presence of FGF1 in rat embryonic fibroblasts and pheochromocytoma PC12 cell line^16,17^. Unexpectedly, no decrease in the protein levels of PUMA, Bax, p21 and TIGAR was seen in etoposide treated COV434-FGF1 cells. They are even significantly more elevated in COV434-FGF1 compared to COV434-Mock cells after 3 or 16 h of etoposide treatment (Fig. 4b, Supplementary Figs. S3, S4).

Finally, we investigated the involvement of p53 transcriptional-dependent activities in etoposide-induced apoptosis in COV434 cells using pifithrin-α (PFT-α), a p53 transcriptional activity inhibitor^39^. A decrease in apoptosis is expected with PFT-α treatment if transcriptional activities of p53 are important for etoposide-induced cell death in COV434 cells. On the opposite, no decrease in the percentage of apoptotic cells (after 16 h of etoposide treatment) was observed upon treatment with PFT-α. PFT-α even slightly increased apoptosis following a 16 h etoposide treatment in COV434-FGF1 cells (Fig. 4c).

The blockage of the transcriptional activity of p53 was confirmed by testing the protein levels of its transcriptional targets (*p21* and *Bax*) in the presence of PFT-α. A decrease of p21 and Bax protein levels was seen 6 or 16 h after etoposide treatment in the presence of PFT-α, confirming its efficiency (Fig. 4d).

In conclusion, p53-transcriptional activity remains unaffected after FGF1 overexpression in COV434 cells and seems dispensable for etoposide-induced apoptosis in these cells.

### The p21 anti-apoptotic activity is not involved in FGF1-induced resistance to etoposide

**Fig. 5:**
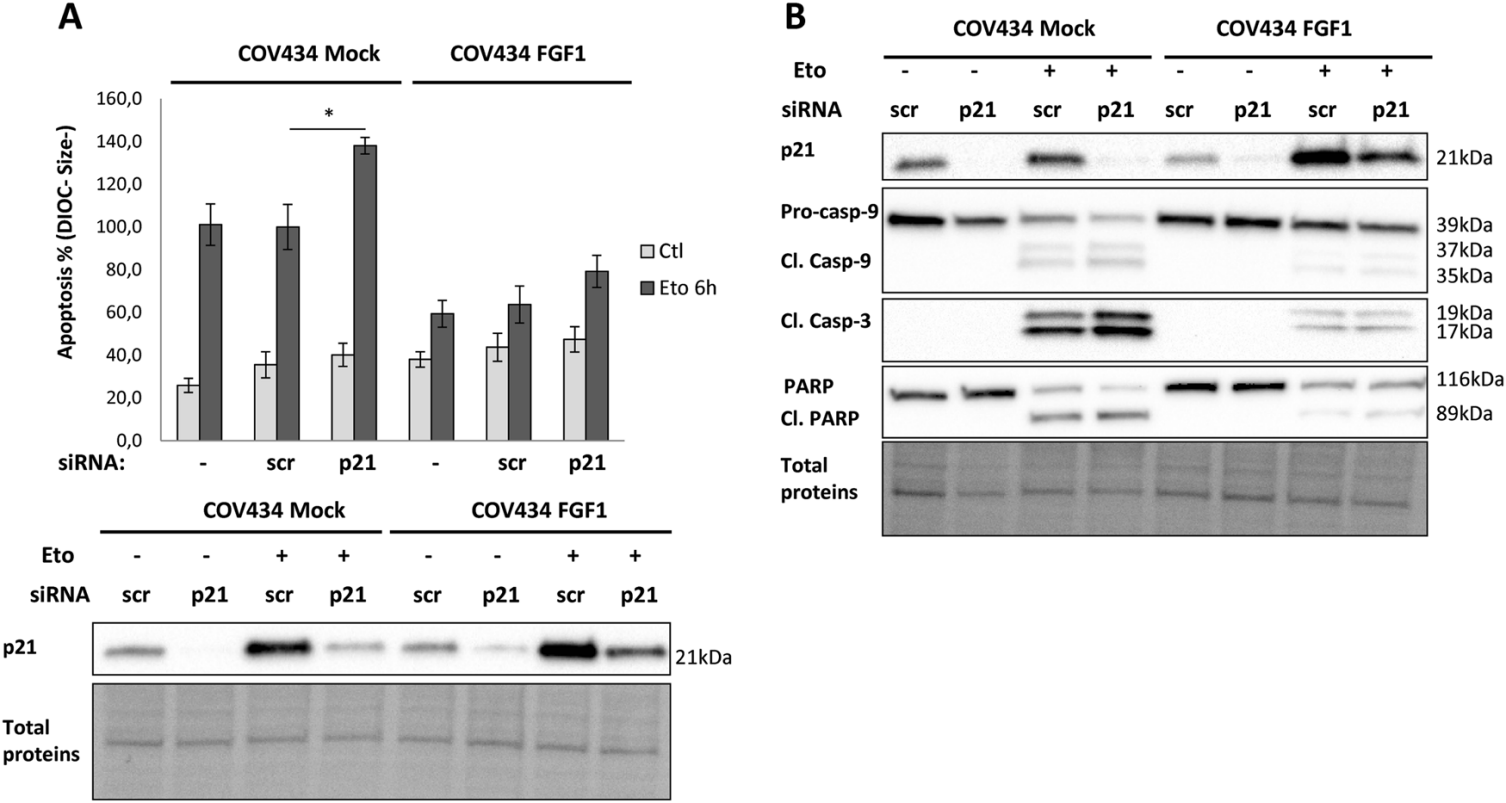
p21 anti-apoptotic activities are not necessary for FGF1-induced resistance to etoposide. **a** COV434 Mock and COV434 FGF1 cells were transfected or not with scramble (scr) or p21 siRNA. Upper panel: average apoptosis rates ± SEM for 2 experiments done in duplicate were measured by flow cytometry in cells treated with etoposide for 6 h, or not treated (Ctl). Lower panel: Western blot analysis for p21 protein levels in COV434 Mock and COV434 FGF1 cells transfected with scr siRNA or p21 siRNA. **b** Western blot analysis of total proteins for p21 and for procaspase-9, cleaved caspase-9, and cleaved caspase-3, and PARP levels. COV434 Mock and FGF1 cells transfected with scr or p21 siRNA were treated or not with etoposide for 16 h. One experiment representative of 3 independent experiments is shown. *P ≤ 0.05, **P ≤ 0.01, ***P ≤ 0.001

As p21 displays anti-apoptotic activities, among others^40^, we examined the significance of the p21 accumulation observed in COV434-FGF1 cells after an etoposide treatment. Our goal was to determine if p21 anti-apoptotic activity was responsible for FGF1 anti-apoptotic activity. Following a transient transfection using a p21 transcript-targeting siRNA or a control siRNA, COV434-Mock, and COV434-FGF1 cells were treated with etoposide for 6 h. In mock cells, we observed a significant increase of the apoptosis rates following p21 knockdown, whereas p21 knockdown showed no significant increase of COV434-FGF1 cells apoptosis (Fig. 5a). Furthermore, we observed a higher rate of cleavage of the pro-caspases-9, pro-caspases-3, and PARP upon p21 knockdown in COV434-Mock cells but not in COV434-FGF1 cells (Fig. 5b).

Altogether these results indicate that p21 exerts anti-apoptotic activities in COV434 cells, but the accumulation of p21 in COV434-FGF1 cells after an etoposide treatment does not seem responsible for FGF1 anti-apoptotic activities.

### FGF1 regulates p53 pro-apoptotic mitochondrial activities

**Fig. 6:**
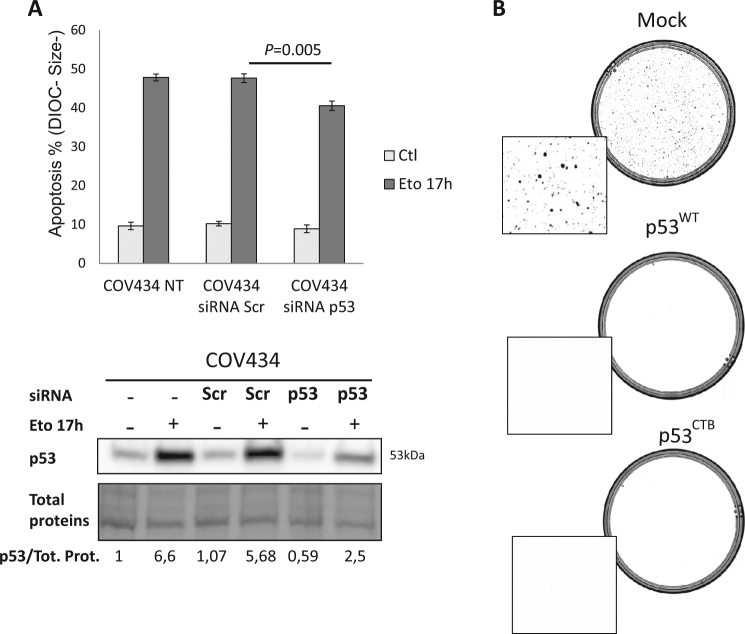
p53 is involved in the induction of apoptosis in COV434 cells. **a** COV434 cells were transfected or not (NT) with scramble (scr) or p53 siRNA. Upper panel: histogram represents average apoptosis rates ± SEM for 2 experiments done in duplicate measured by flow cytometry in cells treated with etoposide for 17 h, or not treated (Ctl). Lower panel: Western blot analysis for p53 protein levels in COV434 cells transfected with scr siRNA or p53 siRNA. **b** COV434 cells were transfected with an empty vector (Mock), a vector encoding p53 wild-type (p53^WT^) and a vector encoding p53 fused to the mitochondrial transmembrane domain of BCL-X_L_ (p53^CTB^). Cells were treated with of G418 for one week and then stained with ethidium bromide and visualized using Chemidoc (Biorad)

Decreasing p53 protein levels with p53-targeting siRNA lowered the apoptotic rate of COV434 cells. This demonstrates the involvement of p53 in the etoposide-induced apoptosis in this cell line (Fig. 6a). Apoptosis rate was slightly decreased in p53 knockdown cells probably due to the remaining high levels of p53 (Fig. 6a). To confirm that p53 is able to kill COV434 cells, clonogenic survival assays were done. COV434 cells were transfected with an empty vector or a vector encoding p53^WT^. After 12 days of selection with geneticin, no clones were obtained for COV434 cells overexpressing p53^WT^. Thus p53 can induce cell death in COV434 cells (Fig. 6b). Given that p53 can exert its pro-apoptotic activities directly at the mitochondrion we tried to determine if mitochondrial p53 is able to kill COV434 cells^41^. Clonogenic survival assays were also done for COV434 cells transfected with a vector encoding p53 fused to the transmembrane domain of BCL-X_L_ (p53^CTB^). p53^CTB^ is predominantly localized at the mitochondria as we confirmed by immunofluorescence (data not shown). As with p53^WT^, no clones were obtained for COV434 cells transfected with p53^CTB^ (Fig. 6b). Furthermore, transient transfections of p53^WT^ or p53^CTB^ showed that mitochondrial p53 is able to induce apoptosis in COV434 cells under basal and etoposide conditions (Supplementary Fig. S5).

**Fig. 7:**
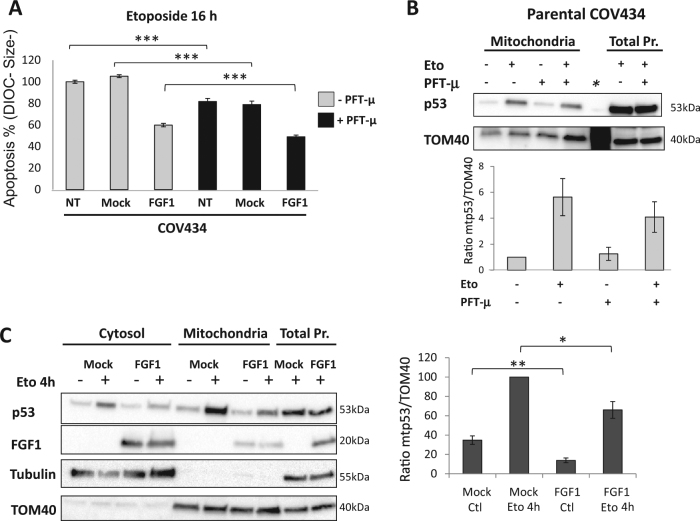
FGF1 regulates p53-mitochondrial localization. **a** Average apoptosis rates ± SEM for 3 experiments done in triplicate were measured by flow cytometry in non-transfected (NT), mock and FGF1-overexpressing COV434 cells treated with etoposide for 16 h. These cells were pretreated or not with the p53 mitochondrial localization inhibitor pifithrin-mu (PFT-µ, 10 µM for 90 min). **b** Non-transfected COV434 cells were pretreated or not with PFT-µ (10 µM for 90 min) prior to etoposide treatment (25 µg/mL for 2h30). Mitochondrial localization of p53 was determined by western blot analysis of enriched mitochondrial fractions (upper panel). Quantification of mitochondrial p53 normalized to TOM40 for four experiments (lower panel). *Molecular weight lane. **c** Cytosolic, mitochondrial and total proteins of COV434-Mock, and COV434-FGF1 cells, treated or not with etoposide for 4 h, were analyzed for p53 and FGF1 localization by western blot (left panel). Quantification of mitochondrial p53 normalized to TOM40 (right panel). Results are from 6 independent experiments, means ± SEM, two-tailed *t*-test results are shown by * for *P* ≤ 0.05, ** for *P* ≤ 0.01, and *** for *P* ≤ 0.001

The involvement of p53 transcription-independent mitochondrial activities in the apoptosis of COV434 cells was also examined by using the p53 mitochondrial localization inhibitor pifithrin-µ (PFT-μ, 2-phenylethynesulfonamide). PFT-µ blocks p53-mitochondrial translocation by interfering with the HSP70/p53 complex formation^42^. While PFT-α was inefficient to inhibit apoptosis upon 16-hour-long etoposide treatment, PFT-µ significantly inhibited the etoposide-induced apoptosis of COV434-NT, COV434-Mock, and COV434-FGF1 cells (Fig. 7a). A decrease of mitochondrial p53 level was observed in the presence of PFT-μ and etoposide compared to etoposide alone. Because of data disparity, this decrease is not statistically significant (Fig. 7b).

To determine whether FGF1 could affect the mitochondrial localization of p53 as does PFT-µ, we next investigated the amount of p53 in the mitochondrial fraction. Without etoposide, mitochondrial p53 level is higher in COV434-Mock than in COV434-FGF1 cells. After a 4-hour-long etoposide treatment, mitochondrial accumulation of p53 was seen in both cell lines. Nevertheless, COV434-Mock still presented higher amounts of mitochondrial p53 than COV434-FGF1 (Fig. 7c). Recombinant FGF1 added to COV434 parental cells also decreased mitochondrial localization of p53 under etoposide treatment (Supplementary Fig. S6). Interestingly, FGF1 was detected in the mitochondrial fraction of FGF1 overexpressing cells (Fig. 7c).

These results suggest that p53 mitochondrial activities are important for etoposide-induced apoptosis in COV434 cells. Furthermore, FGF1 overexpression decreases mitochondrial levels of p53 which could explain the FGF1 anti-apoptotic activity.

## Discussion

Our purpose was to understand the role of FGF1 in chemoresistance in ovarian tumors. Of note, FGF1 is not the sole member of the FGF family that is associated with chemoresistance. FGF2 levels are frequently elevated in solids and hematological cancers. Interestingly expression of FGF2 correlates with resistance of ovarian tumors (among others) to paclitaxel^43^. In addition, FGF2 can even serve as prognostic factor for some cancer types^44^. As FGF1, we observed that FGF2 decreased etoposide-induced apoptosis of COV434 cells (supplementary Fig. S7). Further work will help to determine if FGF2 also affects mitochondrial p53.

In this study, in agreement with FGF1 putative role in chemoresistance, we show that overexpression of FGF1 in the COV434 cell line renders cells less prone to etoposide- and cisplatin-induced apoptosis. Furthermore, we provide new data on the mechanisms involved in apoptotic cell death of these cells. Previous studies had shown that they undergo apoptosis when treated with etoposide or cisplatin and that cisplatin-induced apoptosis partially depends on p53^45,46^. In agreement with these data, etoposide and cisplatin induced the accumulation and activation of p53 in our study. p53 is wild-type in the COV434 cell line^47^ and accumulates at the nucleus (our unpublished data) and mitochondria in the presence of a chemotherapeutic agent. Therefore, p53 could display transcriptional and non-transcriptional activities. Our results using chemical inhibitors targeting these activities suggest that p53 transcriptional activities are rather anti-apoptotic since PFT-α increased cell death induced by etoposide. p21 could be the major actor of this anti-apoptotic activity as we observed that PFT-α strongly inhibits its accumulation. Moreover, p21 knockdown using RNA interference markedly increases etoposide-induced apoptosis.

The p21 anti-apoptotic activity is associated with its cytoplasmic localization which relies on its phosphorylation by Akt^48^. In the cytoplasm, p21 can interact with various proteins involved in apoptosis, including procaspase-3 (for review see^49,50^). Thereby, p21 inhibits procaspase-3 cleavage and subsequent activation^51^. In testicular cancer, cytoplasmic p21 has been associated to cisplatin resistance^52^. Furthermore, recent studies described high levels of cytoplasmic p21 in cisplatin-resistant ovarian cells (C13* cells) and the restoration of sensitivity to cisplatin when this cytoplasmic localization is impaired. The Hsp27 chaperone that is also overexpressed in C13* resistant cells could be responsible for the accumulation of cytoplasmic p21^53,54^. Here, we confirmed the anti-apoptotic activity of p21 upon stress-induced cell death of COV434 ovarian cells. Since p21 can be cleaved by caspases during apoptosis^55^, the accumulation of p21 observed in FGF1 overexpressing cells could be a consequence of the diminished activity of caspases. This could allow for the maintenance of the anti-apoptotic activity of p21. Nevertheless, markedly decreasing overall p21 levels upon RNA interference in FGF1-overexpressing cells leads to a non-significant increase in apoptosis. The p21 anti-apoptotic activity may thus not be the sole or main factor involved in FGF1 anti-apoptotic activities. Another explanation could be that p21 localization is modified by FGF1 and that RNA interference was not sufficiently efficient to decrease cytoplasmic p21 levels in our experiments. Interestingly, we observed a more pronounced accumulation of cytosolic p21 in COV434-FGF1 cells vs. COV434 mock cells upon etoposide treatment (Supplementary Fig. S8).

Only rare data are found in the literature concerning the role of p53 in the apoptosis of granulosa tumor cells. Woods and colleagues showed p53 involvement in cisplatin-induced cell death of COV434 and KGN cells, two cell lines commonly used as models of juvenile and adult GCT respectively^46^. To our knowledge, we are the first to investigate the role of p53 mitochondrial activities during cell death of COV434 cells. We show here that p53 accumulates at the mitochondrion upon etoposide treatment of these cells and that inhibition of this mitochondrial localization using pifithrin-µ decreases cell death. Moreover, as pifithrin-µ, FGF1 diminished p53 accumulation at the mitochondrion. Therefore in COV434 cells, FGF1 affects p53 activities by a novel mechanism, which does not rely on the inhibition of transcriptional activities of p53 such as in neuronal and fibroblast cell lines^16–19^. This is the first report highlighting the regulation of mitochondrial p53 by FGF1.

Besides its regulation of p53 mitochondrial localization, FGF1 could also promote survival through other mechanisms such as regulation of the subcellular location of the oncoprotein MUC1-C. MUC1-C is known to attenuate genotoxic stress-induced cell death, conferring resistance to chemotherapeutic reagents such as cisplatin and etoposide^56^. MUC1-C interacts with activated BAX and inhibits its oligomerization at the mitochondrion^57^. Interestingly, FGF1 can induce mitochondrial localization of MUC1-C^58^. Therefore, one could think that, in COV434-FGF1 cells, MUC1-C could compete with p53 for binding to pro-apoptotic members of Bcl-2 family, thereby inhibiting MOMP.

How FGF1 affects p53 mitochondrial localization must be further investigated. The molecular mechanisms involved in mitochondrial localization of p53 are not fully understood but candidate proteins are TRAF6, Tid1, and CHCHD4. The TRAF6 E3 ubiquitin ligase restricts the mitochondrial localization of p53 in basal conditions^59^. The TRAF6 E3 ligase mediates K63-linked ubiquitination of p53 in the cytosol. Following cisplatin or etoposide treatment, cytosolic p53 is less ubiquitinated by TRAF6 and accumulates at the mitochondrion. Interestingly, *Traf6*^*−*^^*/*^^*−*^ mice cells undergo spontaneous apoptosis in the thymus, spleen and lungs, and the mitochondrial localization of p53 is largely involved in this cell death. It will be interesting to investigate the consequence of FGF1 overexpression on TRAF6 activity in our COV434-FGF1 cells but also in A2780-CIS cells that resist cisplatin and overexpress FGF1 in comparison to A2780 cisplatin sensitive cells^8^. Further work must also be done to determine if FGF1 regulates the mitochondrial import protein Mia40/CHCHD4 and the matrix protein Tid1 that interact with p53 and promote its mitochondrial localization^60–62^.

The precise localization of FGF1 at the mitochondrion must be further investigated as FGF1 has been proposed to interact with the inner membrane protein SFXN1^21^. Mitochondrial subfractionation must thus be done to determine whether FGF1 associates with the outer mitochondrial membrane or localizes inside the mitochondrion (in the inner membrane, the intermembrane space or the matrix). Considering that we and others have shown that p53 and FGF1 can interact^18,21^ and that p53 can localize in the different compartments of the mitochondria^63,64^, it will be interesting to determine whether p53 and FGF1 are found at the same place and interact at the mitochondrion.

The fact that FGF1 was found to interact with the mitochondrial proteins SFXN1 and mthsp70/GRP75/mortalin^21,65^ led us to propose that FGF1 could mediate its survival activities by a direct action at the mitochondrion. Mthsp70/GRP75/mortalin is a chaperone protein predominantly found at the mitochondrion where it participates in the import of proteins in association with TIM proteins. Mortalin is overexpressed in various cancers and can interact with p53 (see ref. ^66^ for a review). This interaction is seen as a mechanism of cytoplasmic retention of p53 to prevent the nuclear activities of this transcription factor. For example, in hepatocellular carcinomas cell lines, mortalin interacts with p53 and its downregulation induces nuclear localization of p53^67^. Furthermore, apoptosis induced by mortalin knock-down is reversed by PFT-µ, suggesting that mitochondrial activities of p53 are inhibited by mortalin^68^. As FGF1 and p53 interact with mortalin and FGF1 regulates p53 mitochondrial localization, one could think that FGF1 may inhibit mitochondrial localization of p53 by promoting mortalin/p53 interaction.

In conclusion, our study provides insights into the mechanisms of cell death in COV434 juvenile granulosa tumor cells and into chemoresistance induced by FGF1. We show for the first time that mitochondrial p53 induces cell death of COV434. Since p53 is not mutated in GCTs, contrarily to most epithelial ovarian tumors, progress in understanding the molecular mechanisms of cell death in these cells is of crucial importance. As GCTs are less frequent than epithelial ovarian cancers (EOC), the study of GCTs has been relatively neglected and therapeutic strategies for EOC have been applied to GCTs^47^. However these tumors greatly differ on both molecular and morphological levels. Investigating the molecular mechanisms involved in response to chemotherapy in GCTs is thus of major interest. Further work must be done to determine if the same mechanisms (p53-induced cell death and FGF1 anti-apoptotic activity) are found in adult (KGN) and juvenile tumor cells. More generally, this study provides the molecular basis for the comprehension of the role of FGF1 in the resistance to chemotherapy in ovarian cancers and could potentially be extended to other cancers overexpressing FGF1.

## Materials and methods

### Cell culture, transfections, and chemicals

The COV434 cell line was a kind gift from Sandrine Caburet. COV434 were cultured in DMEM/F12 medium (ThermoFisher, Waltham, MA, USA) supplemented with 10% FBS (ThermoFisher), 1% GlutaMAX (ThermoFisher), 100 μg/ml penicillin and 100 U/ml streptomycin (ThermoFisher) at 37 °C, 5% CO_2_. COV434-Mock and COV434-FGF1 were obtained following stable transfection with 2.5 µg of pcDNA3.1D V5-His plasmid containing or not the *FGF1* coding sequence, using 8 µl of Lipofectamine LTX and 2.5 µL Reagent plus (Life Technologies, Carlsbad, CA, USA). Transfected cells were selected in 500 µg/mL of Geneticin G418 (Life Technologies) for two weeks. The isolated clones were amplified and used for experiments. For p21 and p53 knockdown experiments, 3 × 10^5^ cells were plated on 6-well plates. At 30% confluence, siRNA transfection was done using 80 pmol of human p21 siRNA, human p53 siRNA or siRNA-A control (Santa Cruz, Dallas, TX, USA) and 4 µl lipofectamine RNAimax (Invitrogen). Etoposide (Sigma-Aldrich, St Louis, MO, USA) was used at 25 µg/mL. Pifithrin-α and pifithrin-µ (2-phenylethynesulfonamide, PFTμ) are from Enzo Lifesciences Farmingdale, NY, USA, and FGFR1/3 inhibitor from Tocris Bioscience, Bristol, UK. Recombinant FGF1 (R&D Systems, Minneapolis, MN, USA) was used in combination with 10 µg/mL heparin (Sigma-Aldrich).

### Clonogenic survival assays

COV434 cells were transfected with an empty vector or vectors encoding p53^WT^ or p53^CTB^ (kindly given by Pr Ute M Moll) as described above for stable transfections. After 12 days of selection in geneticin containing medium, clones were stained with ethidium bromide following the procedure described by Guda et al^69^. Images were acquired using Chemidoc system and the ImageLab software (BIORAD).

### Flow cytometry

To monitor apoptosis, 5 × 10^5^ cells were seeded on 6-well plates. Following the appropriate drug treatment, they were trypsinized, harvested and centrifuged. The cellular pellet was suspended in 0.2 µM of DiOC_6_(3) (Molecular Probes, Eugene, OR, USA) containing culture medium and submitted to flow cytometry after an incubation of 30 min at 37 °C. For cell cycle analysis, the collection procedure was as for apoptosis except that the cellular pellet was suspended in culture medium containing 1 µg/mL of Hoescht 33342 (82261, Sigma-Aldrich) for 20 min at 37 °C. Cytometry experiments were performed on *BD LSRFortessa*™ cell analyzer (BD Biosciences Franklin Lakes, NJ, USA). Analysis was done using the BD FACSDiva software (BD Biosciences).

### Total protein extraction and mitochondrial fractionation

For total protein extracts, 2 × 10^6^ cells were plated on 60 mm dishes. Following an appropriate drug treatment, they were trypsinized, harvested and centrifuged. The cellular pellet was suspended in ELB buffer (250 mM NaCl, 50 mM HEPES, 5 mM EDTA, 0.1% NP40, 0.1 M DTT) supplemented with protease 1/100 inhibitors cocktail (Cat.No.04693116001, Roche, Mannheim, Germany) and 0.2 mM of sodium orthovanadate/phosphatase inhibitors (cat.567540, Calbiochem, San Diego, CA, USA). For mitochondrial isolation, 2 × 10^7^ cells cultured on 100 mm dishes were harvested, centrifuged and washed with PBS. Fractions enriched in mitochondria were obtained either using the Mitochondrial isolation kit for mammalian cells (Thermofisher) according to the manufacturer’s guidelines or following procedure described elsewere^64^. The purified mitochondria were lysed in TBS-CHAPS 2% (EUROMEDEX, Strasbourg, France) supplemented with protease inhibitors.

### Conditioned media and affinity chromatography

Cell extracts and conditioned media from COV434-mock or COV434-FGF1 cells were collected and heparin-sepharose purified as previously described^19^. FGF1 content was determined by western-blot analysis.

### Immunoblotting

Equal quantities of proteins (10 to 30 µg) were run on Mini-PROTEAN TGX Stain Free precast polyacrylamide 4–20% gels (BIORAD) in Tris glycine-SDS buffer (BIORAD). Proteins were transferred on Immobilon-P PVDF membranes (Millipore) in Tris glycine-Ethanol 20% buffer (BIORAD). Stain Free technology allows visualizing total proteins without use of any dye. The following antibodies were used at dilutions from 1/200 to 1/1000: anti-caspase 9 (32539, Abcam, Cambridge, UK), anti-cleaved caspase 3 (5A1E, Cell Signaling, Danvers, MA, USA), anti-PARP (Cell signaling 92845), anti-FGF1 (R&D AB-32-NA, Minneapolis, MN, USA) and anti-FGF1 (Cat.No.010-24161, WAKO, Osaka, Japan), anti-p53 DO1 (Santa Cruz Sc-126), anti-p53pSer15 (Cell signaling 92845), anti-PUMA N-19 (Santa Cruz Sc-19187), anti-Bax I-19 (Santa Cruz Sc-930), anti-p21 C-19 (Santa Cruz Sc-397-G) and anti-TOM40 H-300 (Santa Cruz Sc-11414). Secondary antibodies were HRP coupled (Jackson Immunoresearch, West Grove, PA, USA) and the revelation was performed using Clarity Western ECL Blotting Substrate (BIORAD). Secondary antibodies were HRP coupled (Jackson Immunoresearch) and the detection was performed using Clarity Western ECL Blotting Substrate (BIORAD). The chemiluminescent signal was captured by Chemidoc (BIORAD) and quantification was performed with the ImageLab software (BIORAD).

### Immunofluorescence

1.2 × 10^6^ cells were plated on 60 mm dishes containing coverslips. Following the appropriate drug treatment, cells were fixed with 3.7% paraformaldehyde at 60% confluence then washed with PBS and permeabilized for 30 min in 0.5% Tween20. Nonspecific sites were blocked with PBS-BSA 1% for 30 min. Primary (anti-cytochrome c, BD Pharmingen, Cat.556432) and secondary antibodies (anti-mouse AlexaFluor488, ThemoFischer scientific) diluted in PBS-BSA 3% were incubated for 60 min each. Nuclei were stained with 1 µM TO-PRO-3 reagent (Invitrogen). Mounting was done using ProLong Gold Antifade Mountant (Invitrogen). Image acquisition was performed at the CYMAGES imaging facility (on Leica TCS SPE and Leica TCS SP8 confocal microscopes) and images were analyzed using the ImageJ software.

### Statistical analysis

Two-tailed unpaired Student’s *t*-tests were performed.

## Electronic supplementary material

Supplementary figures
