# Supplementary material for: FGF1 induces resistance to chemotherapy in ovarian granulosa tumor cells through regulation of p53 mitochondrial localization
Source: Oncogenesis. 2018 Feb 21;7(2):18. doi: 10.1038/s41389-018-0033-y (PMC5833868; doi:10.1038/s41389-018-0033-y)
Supplement: Supplementary file 1 — Supplementary figures [file 41389_2018_33_MOESM1_ESM.pdf]

Fig.S1

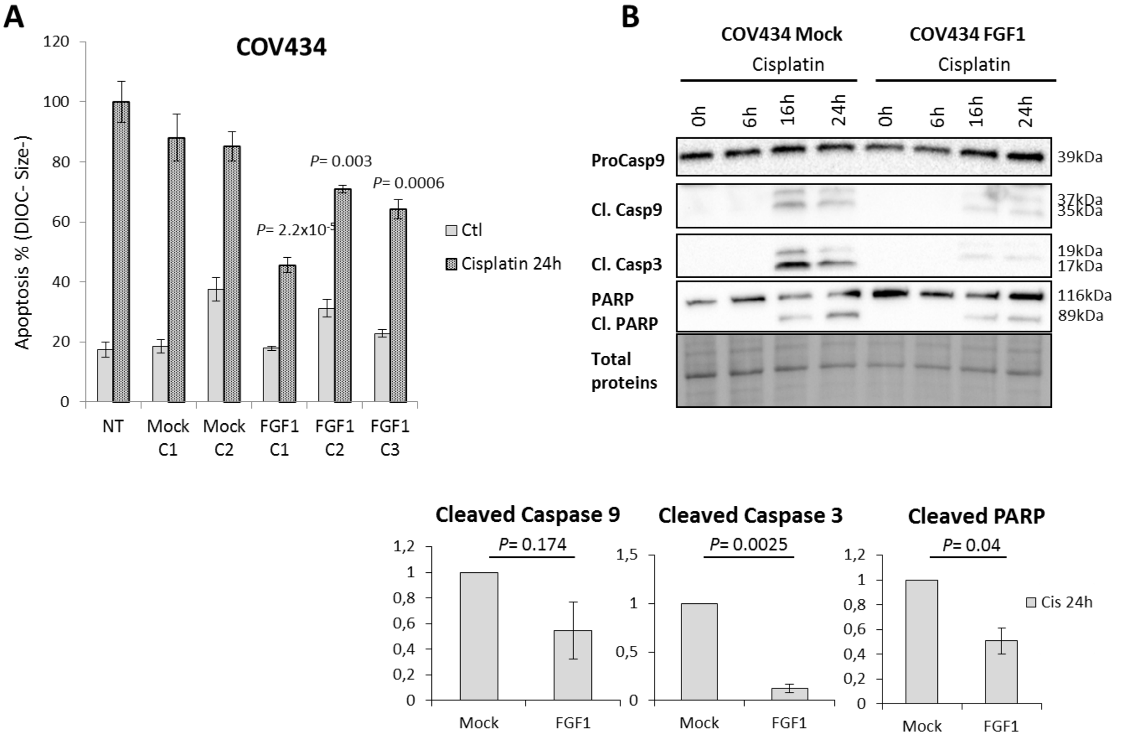

Figure S1. FGF1 overexpression protects COV434 from cisplatin-induced apoptosis.

A) Flow cytometry for apoptotic cells (with low DIOC staining and small size). Non-transfected (NT), Mock and FGF1 overexpressing COV434 cells were treated with cisplatin 7.5 µg/mL for 24h, or not treated (Ctl). N=9, mean ± SEM, T-test compared to NT Cisplatin. B) Western blot analysis of total proteins for procaspase-9, cleaved caspase-9, cleaved caspase-3 and PARP levels. COV434 Mock and FGF1 cells were treated or not with cisplatin 7.5 µg/mL for 6, 16, 24h. Histograms present the fold-change decrease of cleaved caspase-9 and -3 and cleaved PARP in COV434-FGF1. N=3, mean ± SEM, T-test.

Fig.S2

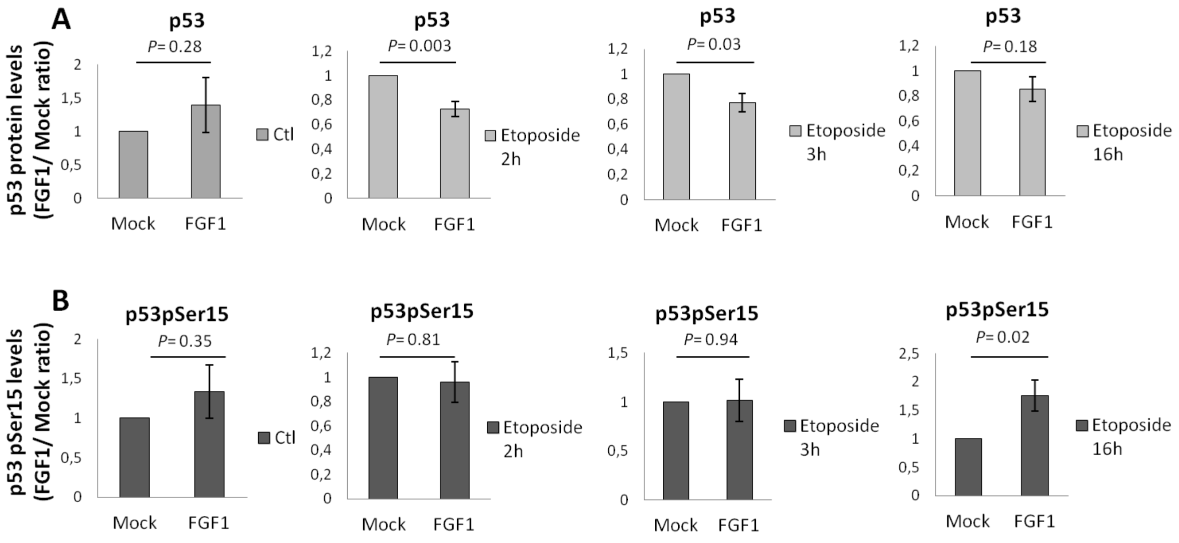

Figure S2. Quantification of p53 and Ser15-phosphorylated p53 levels.

Quantification was realized using the ImageLab software (Biorad). A) p53 protein and B) phosphorylation levels were normalized to total protein levels. Fold changes of p53 and Ser15-phosphorylated p53 levels in COV434-FGF1 cells compared to COV434-mock cells are shown. Mean ratios from 8 independent experiments ± SEM are shown.

Fig.S3

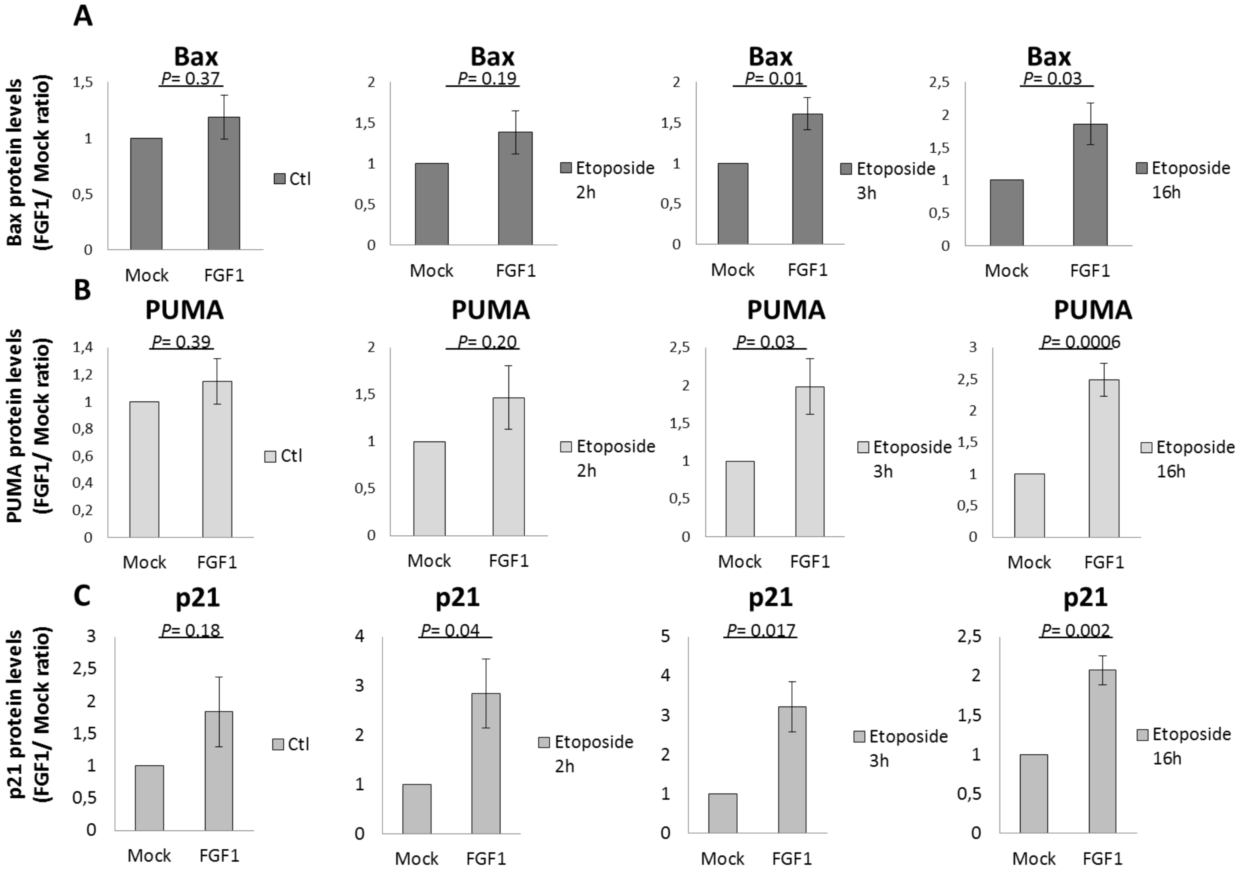

Figure S3. Quantification of Bax, PUMA and p21 levels.

Quantification was done using the ImageLab software (Biorad). A) Bax, B) PUMA and C) p21 protein levels were normalized to total protein levels. Fold changes of Bax, PUMA or p21 levels in COV434-FGF1 cells compared to COV434-mock cells are shown. Mean ratios from 8 independent experiments are shown.

Fig.S4

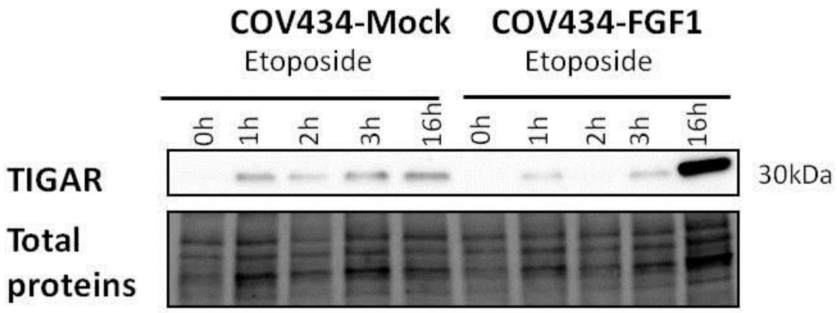

Figure S4. TIGAR protein levels in COV434-Mock and COV434-FGF1 cells.

COV434 Mock and COV434-FGF1 cells were treated or not with etoposide for 1, 2, 3 or 16 hours. Total proteins were analyzed for TIGAR protein levels by western blotting using an anti-TIGAR antibody (Santa Cruz, Sc-166290).

Fig.S5

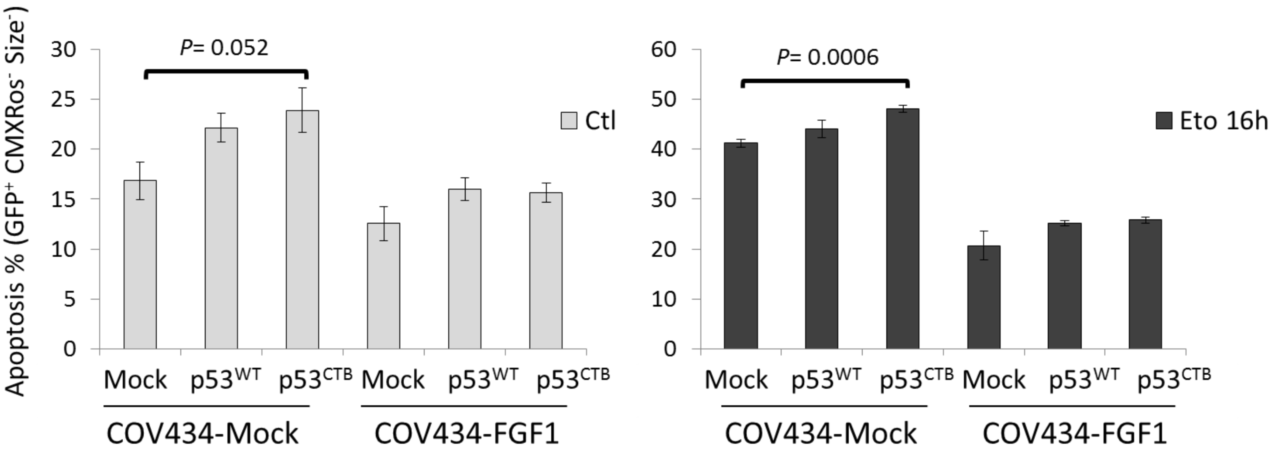

Figure S5. p53 mitochondrial targeting induces apoptosis in basal and in etoposide conditions in COV434 cells.

COV434 cells were transiently co-transfected with a vector encoding GFP along with an empty vector (mock), or a vector encoding $p53^{WT}$ or $p53^{CTB}$. 24 hours following transfection, COV434 cells were treated or not with etoposide for 16 hours. Average apoptosis rates ± SEM for 2 experiments done in duplicate were measured by flow cytometry in mock, in $p53^{WT}$ and in $p53^{CTB}$ expressing COV434 cells in basal conditions (Ctl- left panel) and treated with etoposide (Eto 16h- right panel). Apoptosis rate was determined for the GFP^{+} population. Loss of CMXRos staining and decrease of cell size are the parameters chosen to select for apoptotic cells. CMXRos is a fluorescent dye that stains mitochondria that are not depolarized.

Fig.S6

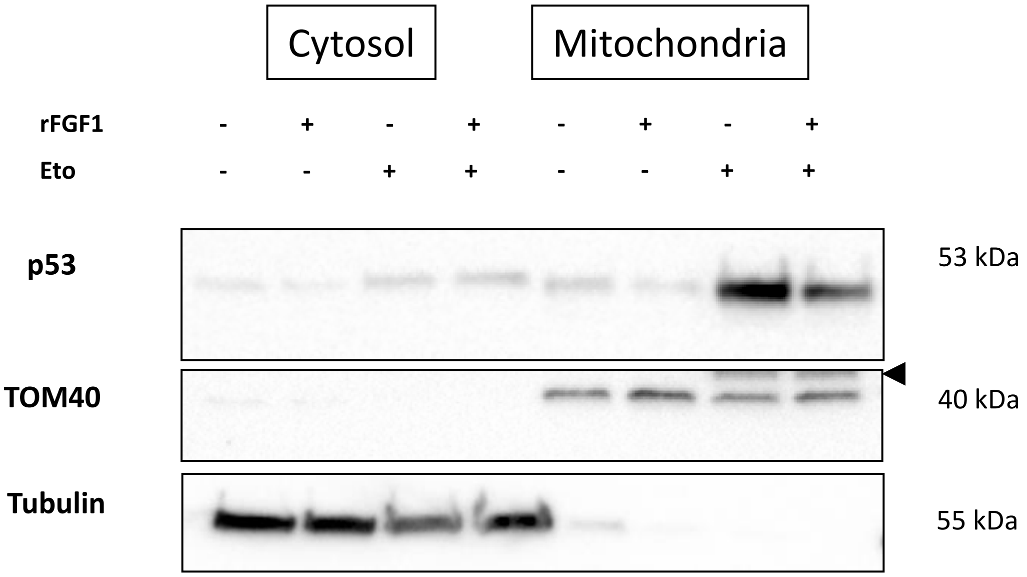

Figure S6: Recombinant FGF1 regulates p53-mitochondrial localization.

COV434 cells were treated or not with human recombinant FGF1 (rFGF1) for 24h prior to an etoposide treatment (25 µg/ml for 4h) or not. Cytosolic and mitochondrial proteins from these cells were analyzed for p53 and FGF1 localization by western blotting. TOM40 and tubulin respectively serve as mitochondrial and cytosolic markers. Bands highlighted by the arrow head correspond to the previous immunodetection of p53 on the same membrane.

Fig.S7

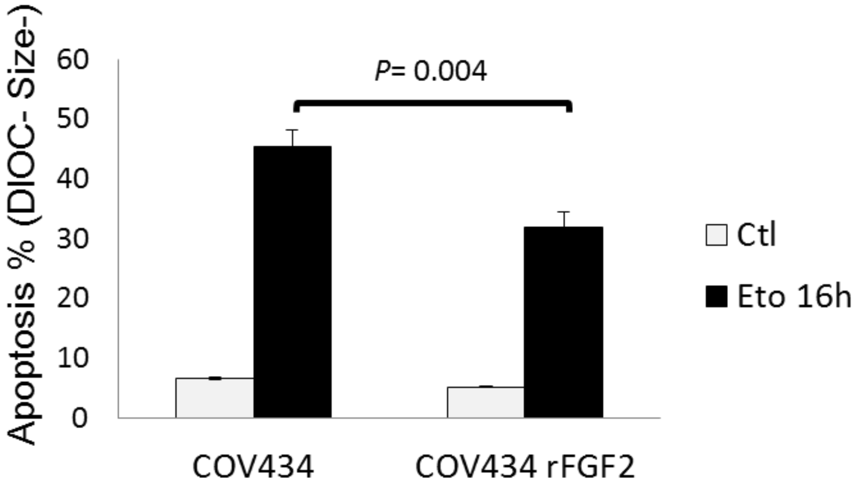

Figure S7. Recombinant FGF2 confers resistance to etoposide-induced apoptosis in COV434 cells.

Average apoptosis rates ± SEM for one experiment done in triplicate measured by flow cytometry of COV434 cells pretreated or not with 20 ng/ml of recombinant FGF2 (rFGF2, *R&D Systems, Minneapolis, MN, USA*) and 10 µg/ml heparin for 24 hours, and treated or not with etoposide (25 µg/mL for 16 hours).

Fig.S8

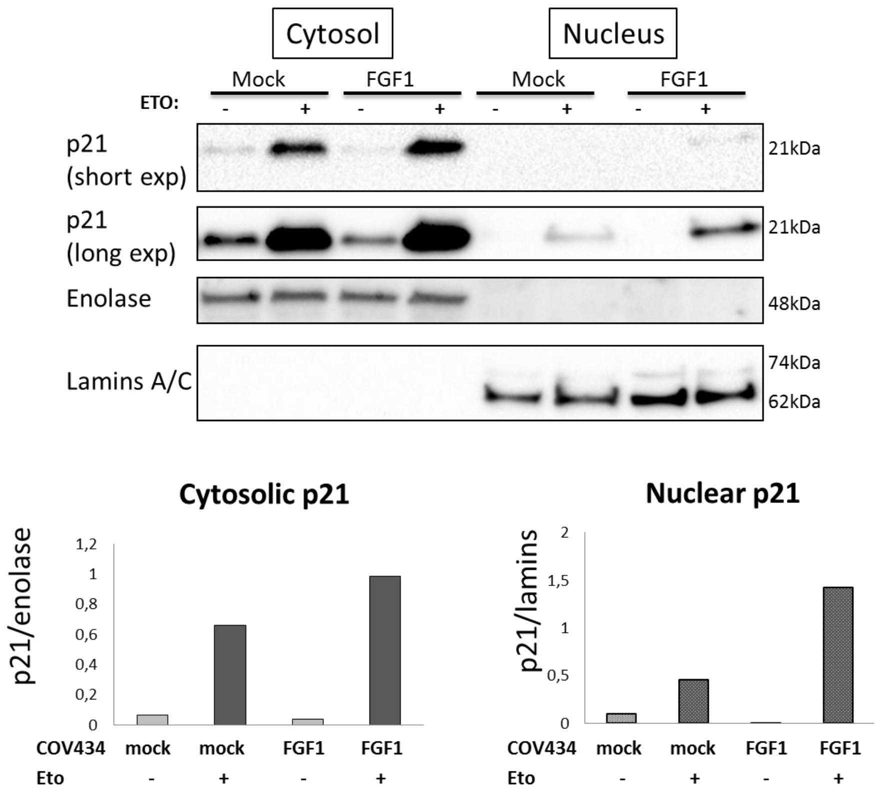

Figure S8. Increased cytosolic localization of p21 in FGF1 overexpressing COV434 cells.

Cytosolic and nuclear proteins were obtained using the NE-PER™ Nuclear and Cytoplasmic Extraction kit (ThermoFisher) from Mock and FGF1 overexpressing COV434 cells treated or not with etoposide (25 µg/ml for 16h). Cytosolic and nuclear fractions were analyzed by western blot to detect p21 (upper panel). The purity of the fractions was confirmed using enolase and lamins A/C as cytosolic and nuclear markers respectively. Lower panel: Quantification of cytosolic p21 normalized to Enolase and of nuclear p21 normalized to Lamins A/C.
